# The Use of Analog and Digital Games for Autism Interventions

**DOI:** 10.3389/fpsyg.2021.669734

**Published:** 2021-08-09

**Authors:** Gray Atherton, Liam Cross

**Affiliations:** Department of Psychology, Edge Hill University, Ormskirk, United Kingdom

**Keywords:** autism, games, intervention, social cognition, emotional intelligence, gamification

## Abstract

Many interventions that target improvements in social communication and other cognitive, learning, and physical issues have been developed to help autistic people. The gamification of interventions offers an alternative approach to fostering and assessing desired behaviors and cognitions in a more naturalistic and emergent setting. In this scoping review aimed at educators, practitioners, and parents of those with autism, we detail studies that have tested game-based approaches to improving the lives of autistic children, adolescents, and adults, focusing on how research into gamification and autism can both progress and can be progressed and implemented. We offer parents, professionals and academics resources to incorporate game-based psycho-educational programs into their current practice.

Autism Spectrum Condition (ASC) is a neurodevelopmental condition that affects approximately 2% of the population. The DSM V (American Psychiatric Association, 2013) which is commonly used for diagnosis describes people with autism as individuals with restricted interests, repetitive behaviors, and social and communicative differences. Autism can be diagnosed using various methods, with the ADI, ADOS, DISCO being the gold standard amongst other diagnostic tools.

Autism was first identified in the mid twentieth century Kanner (1943) and Asperger (1944) through a series of case studies, which described children who showed little interest in social interactions, but high interest in restricted topics. Notably, the children showed atypical interactions during periods of play. For example, rather than building structures with the blocks, a child would use a repetitive motion to move the blocks in recurring ways (i.e., banging them together). When a parent would try and join in the block play, the child would brush their hand away as if their hand was an object rather than belonging to a social agent (Kanner, 1943). Autistic children display atypical play behaviors, tending to prefer independent play, often repetitive, showing less imitation, lacked joint action, and social interaction.

This is notable in that early play behaviors are considered pivotal childhood milestones for several reasons. As discussed at length by Piaget (1997), play behaviors allow a child to engage with emerging cognitive skills. For instance, pretend play enables children to learn concepts such as false belief, and more structured turn-taking board games teach reciprocity and strategy. Crucially, when one is playing with another in a competitive or cooperative game, the experience affords both practice and development of a range of skills, including communication, perspective-taking, emotional regulation, emotional recognition, and sportsmanship. Games also offer children the opportunity to engage in shared attention and joint action with other social agents, as players will imitate other partners' play behaviors to facilitate joint engagement (Eckerman and Stein, 1990). The process of developing joint attention through game-based interactions are even observed across other species (Tanner and Byrne, 2010).

In this way, while a game is ostensibly an enjoyable, entertaining leisure activity, any game, no matter how serious, is simultaneously teaching players how to behave in a group context. Due to in-built rewards systems that track advancement, games may be especially motivating over and above other types of educational interventions (Filsecker and Hickey, 2014). For this reason, it is unsurprising that researchers hoping to improve the lives of autistic people have turned to games when designing interventions.

For example, many autistic people respond differently to social stimulation compared to those who are neurotypical (Chevallier et al., 2012). While typically developed children may automatically imitate a teacher or peer's behaviors, autistic children do not as readily imitate other social actors (Gowen, 2012). They may also be less interested in joining in with a shared activity or remaining focused on a joint goal (Wong and Kasari, 2012). For this reason, the built-in reward system involved in most games (i.e., points, levels, progress bars, feedback) may provide additional incentives. This positive reinforcement may motivate autistic people to continue participating in the game, allowing them to complete the intervention and remain socially engaged with the other players.

Furthermore, games are in and of themselves teaching cognitive and social skills. For instance, a multiplayer game teaches joint attention, turn-taking, strategy, and appropriate social behaviors in response to other players (Rogerson et al., 2018). Some autistic people struggle with these skills and are often late to develop them in line with neurotypicals. Thus, using games, which encourage developing social skills and behaviors, could offer a highly effective interventional format that enables autistic people to improve upon these abilities.

In a broader context, learning and playing games are central to a child's social development in that playing games allows them to form independent relationships with peers (Piaget, 1997). As autistic children often have difficulty forming peer relationships and are more likely to be excluded from social settings (Chamberlain et al., 2007), developing game-play skills may be an essential tool for autistic children to build social capital with peers. Creating opportunities for autistic and neurotypical children to connect in naturally motivating activities like shared enjoyment of a game could serve two essential purposes. First, by playing games in mixed groups, neurotypical and autistic children can learn from one another and build “double empathy” skills, which refers to the ability to understand both neurotypical *and* autistic perspectives (Milton, 2012). Second, autistic and neurotypical children can form reciprocal relationships based on shared interests (i.e., board gaming clubs).

Finally, games are particularly well-suited for customization and need not be overly reliant on outside support. Once children learn the rules of a game, many will be able to interact independently through the game without adult oversight (Lancy and Grove, 2011). The ability to develop independent, peer focused social spheres may be beneficial for autistic children who are often overly reliant on adult assistance, which may interfere with their ability to form friendships (Milley and Machalicek, 2012). Additionally, games are built upon fundamental mechanics (i.e., turn-taking, point collection, random dice throws, card matching, bluffing) that can then be modified to fit a specific theme (i.e., fantasy, space, trains, action-adventure). As most autistic people have restricted interests in a particular domain, they may be particularly interested in games that fit a particular theme. The customization of both analog (traditional board games) and digital (computer and video games) games also makes it possible to design interventions that mainly target specific skills while still providing entertainment.

In sum, playing games are not just childhood fun; they play an essential role in early development and improve cognitive and social functioning throughout the lifespan (Noda et al., 2019). Thus, developing game-based skills, and using games to engage individuals with ASC may be beneficial. Many researchers have recognized this and have developed game-based therapeutic programs that educators, professionals, and families could adopt.

This review will discuss a selection of game-based interventions, which span both analog and digital formats. This scoping review is not intended to offer a meta-analysis or comprehensive systematic review of every use of gamification in autism, but rather provide a pragmatic resource for educators, practitioners, and parents of those with autism who are interested in the use of gamification. The field has not yet as developed to a place where one can definitely state what makes any single or collection of games better than others. As we shall discuss, there is a multitude of issues present in the literature (sample sizes, lack of controls, measurement issues, etc). However, a selection of games are nonetheless identified in order to highlight to practitioners, educators, and parents some of the games that have been utilized in this field, which could be easily implemented in relevant settings and would benefit from further investigation. This scoping review therefore aims to offer an accessible review of some of the existing literature on the use of games in autism interventions to practitioners, parents, and educators and highlight some of the available options. We conclude with our assessment of where game-based research into autism is headed, and how games can continue to improve the lives of people with ASC, including recommendations for game-developers and developmental researchers.

## Methods

To locate relevant research for the literature review, Google Scholar, PubMed, ScienceDirect search engines were utilized. Initial search terms included: Board games, Tabletop games, Mobile games, Video games, Computer games, Games, Game play, Autism, Autistic. These combinations returned a large number of hits, from Google Scholar alone three combinations produced over 1,650,000 returns. The search criteria were therefore restricted to articles published in the last 20 years (from the year 2000 onwards) and the following key, catch-all search terms were used: Autism, Autistic, Games. Searches were performed in the summer of 2020. In addition to these searches, both Ancestry (earlier articles cited by a given article) and Descendary (subsequent articles citing a given article and other papers by authors/labs of a given article) approaches were utilized in combination with key papers.

Articles that were consistent with the overarching aim of this review were isolated. These consisted of articles that offered pragmatic and applicable solutions to the concerns of autistic individuals and their families. Such articles typically focused on interventions utilizing gamification for socio-communicative, or non-social (cognitive, learning, movement) issues faced by autistic individuals and therefore this distinction was made for the purpose of organizing, synthesizing, and reporting findings. All research exploring the therapeutic use of gamification in ASC was sought out, alongside work that explored the use of gamification in assessing aspects of ASC and research exploring autistic interaction with and preferences for games. All relevant studies which used games to improve social or non-social skills amongst autistic individuals were included. Additionally, work which targeted other populations (such as ADHD, Dyslexia) but focused on topics with considerable crossover with and relevance for ASC were also included. Details of all the studies reviewed including their methodology, sample size, and summaries of said studies are reported in Tables 1, 2 split by digital (Table 1) and then analog (Table 2) games. This body of work is first synthesized below, with a focus on how this work can be used in practice categorized by those studies concerning socio-communicative outcomes, and non-social outcomes as well as work looking at autistic players game preferences, in order to provide an accessible resource for practitioners, educators and parents.

**Table 1:** Summary details of studies using digital gamification approaches for autism interventions.

**Reference**	**Participants**	**Methodology**	**Targeted skill**	**Summary**
Silva-Calpa et al. (2018)	7 children with ASC (aged 5–14 years old)	Within groups experiment	Social skills	This article detailed the development and evaluation of “CoASD,” a collaborative game using a touch screen interface. The game was developed to encourage engagement in collaborative tasks. Results showed the game had positive effects on motivating individuals to act with partners and increase an individuals' attention to their partner. Results are difficult to generalize due to the small number of participants.
Ferguson et al. (2013)	8 children with ASC (aged 7–11)	Single case design	Social skills	This article detailed a study showing significant improvements in sportsmanship following the 10-week Nintendo Wii based intervention program. In this study participants played Nintendo Wii in tandem with instructors who modeled appropriate game behaviors and prompted players to display sportsmanship.
Battocchi et al. (2009)	• **Study 1:** • 70, NT (mean age 9.5 years old) • **Study 2:** • 16 children with ASC, • (aged 8–18 years old)	• Study 1 Between groups design • Study 2 Within groups design	Social skills	This article detailed the development and evaluation of “CPG” a collaborative puzzle game for fostering collaboration amongst children with and without ASC. This game was based on traditional jigsaw puzzles and utilized a touch screen interface. Results showed more significant negotiation and coordination amongst those with ASC when the game enforced collaboration. This study was well-powered and provided an interesting example of a puzzle game being utilized to increase cooperation.
Giannaraki et al. (2019)	N/A (not formally tested)	N/A (not formally tested)	Social skills	This article detailed the development of “ADDventurous Rhythmical planet” a 3-D, virtual reality game designed to address social and emotional issues in children with ADHD. Though it focuses on ADHD, it uses both methods and looks at outcomes that could have overlap with ASC. No evaluation of the games is given, only its theoretical underpinnings and development are discussed.
Wainer et al. (2014)	6 children with ASC (aged 8 and 9 years old)	Within groups experiment	Social skills	This article detailed the development and evaluation of a social robot (KASPAR) designed to engage autistic children in social and collaborative play. Over a 10-week proof of concept study, three pairs of children played imitative and collaborative games with KASPAR. Children improved their social behaviors and collaborative skills, which was directly related to their exposure to KASPAR. This study presented an interesting use of a robot partner in studying imitative games to improve social behavior. It is difficult to generalize the findings due to the small number of participants.
Pliasa and Fachantidis (2019)	12 children with ASC (aged 6 and 7 years old)	Within groups experiment	Social skills	This article detailed an evaluation of “Daisy” a socially assistive robot in serious games interventions for collaborative play. Autistic children were found to be more socially responsive when playing games supported by Daisy.
Dautenhahn and Billard (2002)	N/A (theoretical)	N/A (theoretical)	Social skills and imitation	This article detailed the use of “Robota” a humanoid robot doll designed for imitative interaction games in autism interventions and therapy. A theoretical and applied background on the use of interactive robots in autism therapy is discussed, particularly in connection to their usage in developing social skills.
Bernardini et al. (2014)	9 children with ASC (aged 8–14 years old)	Observational study	Social skills joint attention	This article detailed the design and implementation of ECHOES, a serious game, built to help autistic children develop socio-communicative skills. Children interacted with an intelligent virtual agent who was both a peer and a tutor, and who accompanied them while they virtually explored a sensory garden where there are learning activities. While this was a well-powered and controlled evaluation, results showed no consistent increase in social behaviors, although some individuals showed some evidence of partial improvements.
Gallup et al. (2016)	3 individuals with ASC (aged 16–21 years old)	Interviews	Social skills, relationship building, transition into adulthood.	This article detailed a qualitative investigation of mass multiplayer online role-playing games to understand their popularity with autistic online players. Results suggested that autistic players felt that the online environment allowed them to practice skills that generalized to real-life settings, and that it gave them a way to connect with other players socially. They also felt it was helpful to have more transparent social rules in the online environment. This exploratory study's implications are that the popularity of such games with autistic adults may allow targeted interventions within the gaming environment.
Kim et al. (2020)	229 adults with ASC (18–55 years old)	Survey	Social skills and exercise	This article detailed an intervention for autistic adults using a mobile game to increase physical activity. Within the app, users navigate a walk displayed on the screen, and after they follow the correct route, they can solve a puzzle. Scores for users are then shared on leader boards, allowing in-game competition. This app was developed with autistic adults and practitioners. Surveys and focus groups suggested that adult users enjoyed the app and that it promoted physical activity and, through the leaderboard, enhanced kinship to other players.
Abirached et al. (2011)	9 children with ASC (aged 4–11 years old)	Within groups experiment	Emotion recognition	This article details the development and evaluation of LIFEisGAME, an intervention to help with emotion recognition. Children played the narrative-driven game involving creating and becoming an avatar. Results showed that while the children correctly identified emotions post game, this was possibly due to matching instead of recognizing emotions.
Malinverni et al. (2017)	10 children with ASC (aged 4–6 years old)	N/A (not formally tested)	Social skills emotion recognition	This article detailed “Pico's Adventure,” a motion-controlled therapeutic computer game designed to increase foster social initiation, turn-taking, imitation, cooperation, and emotion recognition. The article particularly focused on how to develop an inclusive user and clinician-led game design. Exploratory testing indicated that the game was effective in promoting pro-social behaviors. This presented an interesting application of a user and clinician led approach to developing a motion-controlled game which successfully promoted pro-social behavior in autistic children.
Zakari et al. (2014)	N/A (review)	N/A |(review)	Social skills	This review article detailed 40 serious games designed for improving social behavior, communication imagination, learning, and sensory integration for children with ASD. The article did not formally evaluate the interventions described in the paper. However, it did supply an extensive list of computer games developed for autistic children and the type of equipment that would be needed to run the program in an educational setting. This paper is helpful for professionals looking perhaps to add computer games into an existing program as it mentions what skills are targeted and what resources would be needed.
Narimani et al. (2019)	N/A (not formally tested)	N/A (not formally tested)	Emotional intelligence	This article explores the use of “MSCEIT,” a gamified assessment of children's emotional intelligence. The game was developed using machine learning algorithms to improve the speed and accuracy when assessing children's emotional intelligence. Although an interesting account of the development of gamified assessment of emotional intelligence with clear links to ASC, this work was not directly tested on children with ASC and offered no evaluation of the assessment.
Tanaka et al. (2010)	79 children with ASC (aged 8–15 years old).	Randomized clinical trials	Facial recognition and processing	This article detailed an assessment of the “Let's Face It” intervention for face recognition. Let's Face It features a suite of seven computer games that teach facial emotion recognition skills. The results showed that those who completed the intervention showed reliable improvements in analytic and holistic recognition of faces and features compared to a waitlist group. This study was well-powered and consisted of controlled randomized clinical trials demonstrating advances in facial recognition in autistic children due to a gamified intervention.
Murdock et al. (2013)	4 children with ASC (aged 4–6 years old)	Single case design	Pretend play	This article detailed a study utilizing a tablet story game to improve pretend play skills. In the game, children touched a computer tablet to generate dialogue from the characters, like a digital picture book. Children were then presented with physical toys identical to the characters shown in the story. The intervention showed that following the game, children had a significant increase in their level of play dialogue and play behavior with the toys.
Hoque et al. (2009)	8 children, 5 with ASC (aged 8–19 years old)	Between groups experiment	Speech production	This article detailed a suite of computer games designed to help speech production. Various assessments were reported, and results suggested the games provided a useful and engaging language learning platform.
Hiniker et al. (2013)	N/A (not formally tested)	N/A (not formally tested)	Therapy addition	This article detailed the creation of “Go Go Games” a suite of therapeutic games for young children with ASC. It was designed to complement existing practitioner-led therapy to increase the total therapy time for those who needed it. The suite of games is based on pivotal response treatment, and focuses on improving key behaviors that have wide-ranging implications, such as responding to cues. Thirty autistic children (ages not reported) were involved in designing the three games in this suite. This study provides an interesting, evidence-led approach. Although released on the app store, very little evaluation of the game is given.
Whalen et al. (2010)	47 children with ASC (aged 3–6 years old)	Between groups experiment	Language and cognitive abilities	This article detailed an experimental study of TeachTown, a computerized game developed to improve language and cognitive abilities. During normal school hours, children took part in Teach Town for 20 min a day over 3 months. Teachers involved in Teach Town supplemented classroom lessons with Teach Town curriculum. Compared to controls, participants who used TeachTown improved on language and cognitive measures, and those who used it more had more considerable gains. This study presents a well-powered and controlled example of a digital game bootstrapping language and cognitive abilities in those with ASC.
Li et al. (2018)	65 children 33 with ASC (aged 2–17 years old)	Between groups experiment	Executive functioning	This article detailed the creation and validation of a mobile gaming app used to assess executive functioning in autistic children. Three games were developed for use with a tablet, and they tested the executive functioning constructs shifting, short term memory, and inhibition. Results showed that autistic children played the game differently, revealing core differences in executive functioning across groups. Game results also showed that performance on all three games correlated with age, and the skill shifting correlated with IQ.
Mercado et al. (2019)	12 children with ASC (aged 4–11 years old)	Within groups experiment	Sustained attention	This article detailed the development and testing of a neurofeedback brain training game using EEG called FarmKeeper. Sixty autistic children participated in a collaborative development process to develop the game, designed to stimulate sustained attention. The results showed that participants improved attention and found the game fun and user friendly.
Davis et al. (2007)	6 children with ASC (aged 5–7 years old)	Within groups experiment	Narrative construction	This article detailed a within-subjects study evaluating TouchStory, a computer game involving ordering different parts of a story into the correct position. TouchStory was designed to help children practice narrative comprehension. Results suggested that the children significantly improved their ability to construct narratives. It is difficult to generalize these results due to the small number of participants.
Behnamghader et al. (2019)	N/A (not formally tested)	N/A (not formally tested)	Reading	This article described a gamified reading intervention for children with dyslexia based on a modified version of “Mario.” This game was developed for children (aged six to eight) with dyslexia to increase motivation and participation in reading and has not been used with ASC. However, it may offer an interesting tool for developing reading skills in the population.
Edwards et al. (2017)	30 children 11 with ASC (aged 6–10 years old)	Within groups design	Motor skills	This article detailed a study in which children played a sport active video game. Improvement in both perceived and actual movement skills were assessed. Results showed that while actual skills didn't improve in either group, perceived skills improved in the ASC group. This study is particularly interesting as it indicated that amongst autistic children, movement-based games may bolster more confidence in physical abilities.
Politopoulos et al. (2019)	N/A (theoretical)	N/A (theoretical)	Motor skills	This article detailed the development and initial evaluation of “Magic-Matt” a movement-based games intervention to aid motor skills development. This paper offers a theoretical overview of exergames, natural user interfaces, and serious games in the context of interventions. Though not focusing specifically on ASC, there are clear overlaps and cross over considering the motor aspects of ASC.
Khaleghi et al. (2019)	N/A (not formally tested)	N/A (not formally tested)	ADHD assessment	This article detailed the development of the gamification of an assessment tool for ADHD. While not focusing on ASC, there is relevant overlap between ADHD and ASC and a considerable degree of comorbidity. Therefore this work offers valuable insights, though this development is yet to be evaluated.
Brown and Murray (2001)	N/A (review)	N/A (review)	Play in interventions	This article detailed a useful summary of differences in the way's children with and without an ASC diagnosis engage in play. It also highlighted critical play behaviors that should be targeted through a play intervention. The paper gives a good overview of the importance of play and suggests strategies for successfully incorporating play into ASC interventions and interactions.
Gaudi et al. (2019)	N/A (theoretical)	N/A (theoretical)	Serious games	This Master's thesis discussed the development of a serious game framework to help clinicians develop games for children with ASD. As children with ASC may have particular sensory needs or specific restricted interests, it can help individualize an intervention to reflect these preferences. In this paper, the author discusses creating a serious game and how therapists can use the simple interface to create more gaming interventions for specific clients.
Grossard et al. (2017)	N/A (review)	N/A (review)	Game design and playability	This review article focused on elements inherent to game design and playability for autistic players. It reviewed 31 serious games designed to foster better social abilities (split into emotion recognition and social skills) in children with ASC. This review highlighted a promising body of findings but with the need for games targeting those with more severe needs/lower functioning and the need for better evaluation of games.
Mazurek et al. (2015)	58 adults with ASC	Survey/Interviews	Video game preferences and experiences	This article explored the video game preferences and outcomes of autistic adults. Findings highlighted stress relief, immersion, and social connection as positive outcomes to playing video games in the population, though addiction and negative social interactions were also highlighted. In terms of motivations and preferences in games achievement, graphics, story and creativity were found to be essential elements. This study presented interesting observations on the video game preferences of those with ASC and highlighted the positive effects of videogame play.

**Table 2:** Summary details of studies using analog gamification approaches for autism interventions.

**Reference**	**Participants**	**Methodology**	**Targeted skill**	**Summary**
Daubert et al. (2015)	2 children with ASC (9–10 years old)	Single case design self-reports	Social skills	This article detailed an intervention based on power cards, a visual technique capitalizing on an individual's motivation and interests to teach skills or encourage behaviors. Power Cards were used to develop social initiations and pro-social behavior during three games (Topple Operation and Honey Bee Tree). Power cards depicted appropriate behavior in problematic situations in order to capitalize on an individual child's restricted interests to foster social communication and turn-taking. Results showed that turn initiation and relinquishing (but not commenting) increased following the intervention.
Jung and Sainato (2015)	3 children with ASC, 6 NT children (aged 5–6 years old)	Single case design	Social skills	This article detailed a study where off-the-shelf board games were personalized for autistic children based on their special interests, which included creating personalized video modeling stimuli. Games played included Candy Land and Make n' Break. Researchers assessed whether social engagement with peers, non-verbal attention, and inappropriate behavior changed during the intervention using video coding. Results showed a significant increase in positive behavior and decreased negative behavior following the intervention.
Klopotova and Krupnova (2020)	6 children with ASC (aged 4–8 years old)	Within groups experiment	Social skills	This article detailed an experiment using two board games “Walker and Memory.” Attention to partner and reciprocal communication improved over 10 weeks across 40 gaming sessions. This study's effects are difficult to generalize due to the small number of participants.
Fein (2015)	Adolescents with ASC who attended Journeyfolk camp (ages not specified)	Ethnographic field study	Social skills Perspective-taking Mental abstractions	This article explored the experiences of autistic adolescents at a summer camp dedicated to live-action role-playing games and table-top roleplaying games like Dungeons and Dragons. Using observations from fieldwork, the author concluded that these escapist games allowed the campers to construct narratives about their autism diagnosis that were shared and valued. Shared interests in the games were sources of power, strength and promoted acceptance.
Baker (2000)	3 children with ASC (aged 5 and 6 years old)	Single-case design	Social skills Joint attention	This article detailed an intervention based on a “Bingo” game to increase sibling play by incorporating ritualistic activities into gameplay. When taught a play intervention utilizing this ritualistic behavior, joint attention, positive affect and social interactions of autistic children increased. Repetitive behaviors also decreased. The effect was maintained across 3 months, and the effects generalized to other settings.
Carvalho et al. (2015)	N/A (not formally tested)	N/A (not formally tested)	Social skills Emotion recognition	This article detailed the development of a mobile phone game for autistic children to strengthen their emotion recognition abilities. In the game, which operated through a digital picture book, children interacted with a boy named Tobias, who had various experiences like going to a zoo or a party. Within the game, children had to match which facial expression Tobias would display based on the story's context. The intervention was not formally evaluated.
Katō (2019)	59 children with ASC (aged around 14 years old)	Within groups experiment	Quality of life outcomes	This article detailed two studies in which autistic adolescents participated in tabletop role-playing games. Researchers found that following the intervention, participants improved their emotional well-being and friendships. This study was well-powered and a pertinent example of how role-playing games may have the ability to improve quality of life.
Dell'Angela et al. (2020)	177 NT children (aged 8–12 years old)	Between groups experiment	Emotional competency	This article detailed an experimental study in which children were assigned to play four sessions of either modified board games developed to help emotional competence, or off the shelf control games. Results showed that children with higher emotional competency found the emotional recognition and differentiation games less difficult, and all children reported enjoying the games to the same degree as those off the shelf. Though not directly focusing on ASC, this paper showcases how to modify existing board games to develop emotional competency and provides an adequately powered and controlled experiment showing these games can be just as enjoyable as commercially available off the shelf games.
Davis-Temple et al. (2014)	3 children with developmental delays and 3 NT children (aged 4 and 5 years old)	Single case design	Gameplay performance	This article utilized games which required participants to roll die, match colors, and move pieces. The experimenter used prompting in line with applied behavioral analysis to assist the children in learning the steps to the game and initiating appropriate social responses to gaming partners. Results showed a significant increase in independent board gameplay following the intervention. Though not directly focusing on ASC this paper showcased how to break down steps of games and teach them to those with developmental delays.
Oppenheim-Leaf et al. (2012)	2 children with ASC (aged 5 and 7 years old)	Single case design Observation	Game learning	This article detailed an intervention utilizing three different structured board and card games (Go Fish, Yahtzee junior, and Uno) with two children with ASC. Both participants successfully learned all three games and could generalize gameplay behaviors to other opponents and different situations in less structured sessions. This approach focused more on autistic children's ability to learn a game and generalize that learning to other situations than any benefits of actually doing so.
Satsangi and Bofferding (2017)	10 children with ASC (aged 4–10 years old)	Between groups experiment	Math ability	This article details a replication of earlier research showing linear board games can improve numerical skills in autistic children. Children played a board game involving rolling a die to progress on a track. Half the children played with a focus on numbers of tiles on the track, the other half on the color of tiles. A greater understanding of numerical relationships was demonstrated by those who had played the game with a focus on numbers rather than shapes. This study represents a well-controlled demonstration of a simple, novel board game utilized to improve numerical math ability in children of varying ages.

## The Review

### Socio-Communicative Outcomes

The social aspects of autism are among the most widely researched and focus most prominently within targeted interventions. Many gaming interventions for those with ASC also concentrate on developing social skills, though the mechanisms used to improve these skills vary.

Several gaming interventions, particularly with younger children with ASC, use behavioral approaches embedded within game-play to increase social responsiveness and scaffold social development. For instance, Daubert et al. (2015) used Power Cards, small double-sided index cards, to improve game-based behaviors in young children with ASC. The Power Cards featured on one side the autistic child's favorite character, while the other side described how that character would optimally behave when playing board games (such as having good sportsmanship and encouraging other players). For instance, one participant interested in the Ninja Turtles had Power Cards written from the perspective of one of the turtles who demonstrated appropriate play behavior (i.e., the Ninja Turtle Donatello telling his friends “You did it!” and “You won!”). Participants viewed these cards at the beginning of the game-play session, and when needed, were prompted to review the cards when they needed reminders. Results showed that the participants significantly improved their ability to initiate and relinquish a turn following the intervention.

Several studies also developed games that used modeling combined with behavioral reinforcement. Ferguson et al. (2013) used Nintendo Wii Baseball to teach six children with ASC sportsmanship skills over ten sessions in an outpatient clinic. Instructors first modeled appropriate game-play behaviors such as taking turns and giving a compliment post-game and then awarded points to players who engaged in the behaviors. Jung and Sainato (2015) also used modeling in their intervention with slightly younger children with ASC. Borrowing from the Power Card method, children's special interest characters (i.e., a princess) featured in video recordings of adults modeling appropriate game-play behaviors. Children first watched the videos before playing board games like Candy Land, and then used them as references when they needed prompting. When the children engaged in appropriate behaviors during the game, they were rewarded with tokens. Both studies found behavioral reinforcement and modeling led to increases in appropriate behavior. Jung and Sainato (2015) also found increased engagement with peers and generalization of learned skills to a novel game.

Central to both the Jung and Sainato (2015) and Daubert et al. (2015) studies was the incorporation of restricted interests to enhance game-based motivation. Another study that utilized this approach was Baker (2000). In this intervention children with ASC were encouraged to develop game-play behaviors by playing pre-existing games tailored to reflect their unique special interests. For instance, one boy had a preoccupation with crashing toy cars together. To incorporate this into a game, the researchers devised a version of Bingo that involved choosing toy cars to launch off a ramp and crash, thus triggering a Bingo tile to be called. Using a single-case design, the researchers demonstrated that, following from baseline, the three children tested showed significantly improved play-based behaviors, which transferred to games that were not based on special interests.

Thus far, the projects discussed relied upon existing, “off the shelf” games and modified them to include behavioral conditioning, modeling, and special interests. Other studies instead created new games to teach social constructs explicitly. For instance, several games used narrative storytelling to teach children with ASC socio-communicative skills. Tobias in the Zoo (Carvalho et al., 2015), TouchStory (Davis et al., 2007), and iPad play story (Murdock et al., 2013) all used mobile gaming technology to encourage children with ASC to interact with virtual “storybooks.” In Tobias in the Zoo children interacted with an avatar, Tobias, who experienced different scenarios (i.e., a zoo visit, a birthday party) which caused him to experience various emotions. To win the game, the child needed to correctly identify Tobias' feelings at various points in the story. TouchStory consisted of autistic children dragging story panels, which were pictures showing sequential story scenes, into the correct position relative to one another. In the iPad play story, pairs of autistic children read a story together on an iPad about various characters experiencing certain events (i.e., firefighters in a fire truck going to rescue a girl from a treehouse). After reading the story, the children then interacted with toy versions of the story characters and were encouraged to re-enact the story through symbolic play. While Tobias in the Zoo requires formal testing, children's narrative comprehension following TouchStory showed some improvement. Following the iPad play story, children demonstrated the ability to use the narrative they read on the iPad as the basis for their reciprocal pretend play (Murdock et al., 2013).

Several games explicitly focused on emotion recognition, an area of delayed development for autistic individuals, to target improvement. In Life is Game (Abirached et al., 2011), autistic children picked a custom avatar and then identified the avatar's emotional expressions. There were also options for making the game more challenging by hiding the eyes or mouth. Let's Face It (Tanaka et al., 2010) consisted of seven computer games that encouraged different facial recognition skills, including recognizing facial identities, emotions, and holistic processing of eyes. After 20 weeks, autistic children showed improved face recognition (such as holistic processing of the eyes) but did not improve all targeted skills (such as facial identity recognition).

Rather than explicitly teach socio-communicative skills, some games embedded them within the game's mechanics. Dell'Angela et al. (2020) modified three existing board games already popular with children to target specific emotional competence skills. For instance, the researchers changed the game Code Names (a game where players must give clues to their teammates to link target words) so that rather than pick any word as a clue, players instead had to pick an emotional word as their clue. In a large sample of typically developed children, the researchers found that children with higher emotional competence skills were the most successful at the game and found the game most accessible. In another study, Bernardini et al. (2014) created the computer game ECHOES in which autistic children interacted with an avatar in a magical garden in a way that supported the use of certain behaviors. For instance, embedded within game-play were cues that encouraged joint attention and symbol use. While these behaviors frequency was not directly assessed, the children became more socially responsive to practitioners throughout multiple gaming sessions.

While many games focused on improving socio-communicative abilities in autistic individuals, several took a different approach. Rather than behaviourally reinforcing behaviors or explicitly teaching individual social skills, some interventions used games to more indirectly encourage social communication between players. For instance, Wainer et al. (2014) developed an imitation game to be played with two players with ASC and KASPAR the robot. KASPAR was a humanoid animated doll that could verbally and physically interact with humans. Using a digitized version of Simon Says, researchers found that the children spent more time interacting with one another when playing the game with KASPAR than when playing with only one other.

In Pico's Adventure (Malinverni et al., 2017), children interacted with an avatar, parents, and peers in a virtual environment where they must complete challenges to assist Pico the alien, an animated character. Exploratory results showed that through engagement with the task, autistic children were more expressive and directive with one another within game-play. Finally, autistic children were tested on their ability to interact with one another when playing the Collaborative Puzzle Game (Battocchi et al., 2009), which was presented on a digital tabletop and required players to move digital puzzle pieces simultaneously with a partner. Results showed that players who were required to collaborate in this way were more coordinated and engaged in more complex interactions.

While most of the games previously discussed cater to children, several games have been beneficial to autistic adolescents and adults for socio-communication skill development. In an ethnographic study, Fein (2015) spent time at a summer camp for adolescents with ASC where they spend time engaging in Live Action Role Playing and tabletop role-playing games like Dungeons and Dragons. Fein (2015) found that the games were incredibly engaging for campers. It allowed for structured social interactions between players specific to the game and promoted a narrative of inclusion and acceptance within the games' stories. Katō (2019) tested the effect of tabletop role-playing games on improvements in social speech and changes in quality of life in adolescents with autism following either four or fourteen sessions of tabletop role-playing games. For the four participants who played fourteen sessions, socio-communicative skills improved following the intervention. For the children who played four sessions, total scores on a quality of life measure significantly increased.

Qualitative research on online games for autistic adults also suggests that they can improve socio-communicative skills and quality of life. Mazurek et al. (2015) found that autistic adults spent more time on average playing video games than neurotypicals. They experienced distinct social rewards from video game-play, including forming friendships with the video gaming community and relief from social stress. Gallup et al. (2016) also found that in addition to forming friendships with others in massive multiplayer online communities, autistic adolescents reported socio-communicative improvements in online settings where they could practice skills in safe spaces. They also reported an improvement in their ability to use online communication strategies. Full details of the studies discussed here can be found in Table 1 (digital games) and Table 2 (analog games).

### Non-social Outcomes and Game Preferences

Individuals often experience challenges in other life areas outside of socio-communicative domains, including academic and physical difficulties. Several studies targeted improvements in these specific domains through the use of games. We now turn our attention to work exploring non-social outcomes of gamified research in ASC and autistic game preferences.

TeachTown (Whalen et al., 2010) is a computer-assisted intervention designed to teach young autistic children social and academic skills through an online curriculum. TeachTown is delivered to children daily and utilizes pivotal response training to reinforce correct responses through verbal praise and graphics. In a randomized control trial, children who received TeachTown instruction showed improvement compared to those in the control group on a standardized vocabulary measure. They also significantly improved their scores from baseline, and those who spent more time in the program showed the most improvement. Satsangi and Bofferding (2017) designed a simple board game to improve the numerical knowledge of autistic children by teaching them to roll dice and move tokens along a colored number line. Results showed that across the 10 participants, the ability to make numerical estimates significantly improved amongst those who practiced matching numbers rather than colors. Finally, many autistic individuals have difficulty with prosody or speech production. Hoque et al. (2009) developed a computerized speech therapy game that focused on improving a player's speech intelligibility. Across a suite of games, results suggested that in eight children, five of whom had an ASC diagnosis, language learning improved.

Many autistic individuals experience motor difficulties, including reduced coordination and reduced physical activity levels than those with typical development. To improve the physical capabilities of autistic people, several active games have been modified or created to meet the community's needs. For instance, Edwards et al. (2017) had children with and without ASC engage in a Nintendo Wii program using several sports-related competitive games for 6 h over 6 weeks. They found that neither ASC nor NT children improved on objective measures of object control (i.e., throwing, kicking, and catching a ball). However, the ASC group significantly improved their perception of their object control competencies, indicating Nintendo Wii games can improve sports-related confidence. PuzzleWalk was an intervention created to improve the physical activity levels of autistic adults (Kim et al., 2020). To complete a puzzle, adults walked in a particular pattern displayed on their mobile phones. Preliminary results showed that it was seen as user-friendly and engaging and could be a useful way to encourage physical activity in the adult ASC community.

Individuals with ASC also experience difficulties maintaining rhythm, which some hypothesize may account for some of the social differences observed in the population (Trevarthen and Daniel, 2005). Though not initially developed for children with ASC, but instead children with ADHD, Giannaraki et al. (2019) developed the game ADDventourous Rhythmic Planet, a game that is played in virtual reality in which players use a drum to create rhythm. In this story-based adventure, the hero is an alien that continues onto the game's next stages if the player reproduces a rhythm correctly. The game also progresses from single to multiplayer, encouraging coordinated movement with peers. In another game, Magic Mat, Politopoulos et al. (2019) created a mat that can track movement and guide on-screen actions. In their study, users could play a whole-body form of Tetris in which moving on the mat guided blocks to fall in corresponding gaps at the bottom of a large video screen facing the player. Though both ADDventurous Rhythmic Planet and Magic Mat have not been formally tested, nor were they explicitly designed for children with ASC, both tap areas of need (for examples of movement and rhythm problems in autistic individuals see, Marsh et al., 2013) in novel ways through the use of unique technologies.

### From Research to Practice

A primary aim of this work is to highlight and provide access to these interventions so that they can be more widely adopted. To achieve this, we have provided several resources intended to synthesize and categorize relevant research in the way most useful to practitioners, based on the skills they target and the equipment and materials needed to implement them. A summary of relevant work is therefore divided into analog and digital games and summarized in two tables. Table 1 presents research on the effects of digital games, and Table 2 the effects of analog games, on the social and non-social development of children with ASC. With the exception (in both cases) of several studies using games with typical and other special populations, all of which tap skills that are relevant to ASC. In these tables, we detail the project, the participants, the methods, and the usability of the project. These resources are intended to give an overview of relevant game-based autism interventions that have been developed and tested, rather than an exhaustative list.

## Digital Games

### Example Digital Games

TeachTown (Whalen et al., 2010) is an excellent example of how digital games can be formally tested and incorporated into ASC therapeutic curricula. In this computer game, children with ASC completed daily online challenges, teaching them academic and social skills lessons. Children who participated in TeachTown were compared to waitlisted children, providing a much-needed control condition. Two types of data were collected, scores on cognitive assessments pre and post-intervention, and scores within the TeachTown game which tracked daily progress. Finally, teachers who participated in TeachTown incorporated TeachTown materials into their lesson plans, ensuring that TeachTown concepts were not learned in isolation but retained through different forms of engagement (i.e., a blended learning model). Such measures and supplementary materials provided much-needed rigor and structure to the intervention, which would be helpful if adopted by similar digital gaming interventions.

The Collaborative Puzzle Game in Battocchi et al. (2009) and the imitation game with KASPAR the robot (Wainer et al., 2014) are also examples of how digital technology can enhance gaming interventions for ASC. Though the technology used in these interventions is not yet commonplace (KASPAR is still a prototype), they are examples of digital gaming interventions that may become commonplace in future autism interventions. Importantly, both studies demonstrate how relatively simple games (puzzles, Simon Says) can be modified to be more technologically complex while still retaining the integral elements of game play.

For instance, in the Puzzle Game, which used a Diamond Touch table, players completed jigsaw puzzles with a partner. In the experimental condition, players could not move a puzzle piece unless it was also being touched simultaneously by another player, meaning they had to work in synchrony to move the pieces around the table. By analyzing the game log, the researchers found that the additional need for synchrony with a partner increased the level of interaction between players, and led to more problem-solving (it is worth noting that synchronous movements have been shown to foster a wide range of prosocial outcomes amongst those who take part including, rapport, cooperation, and a reduction in prejudice), for a review see Cross et al. (2019). This is an excellent example of how a digital tabletop's unique capabilities can be maximized to improve an existing game design (i.e., a collaborative puzzle). Battocchi et al. (2009) also demonstrated the improvement that a digital game affords with regards to progress tracking. By having the puzzle activity presented digitally, the researchers could effortlessly record every move made by the players, which allowed for a fine-grained analysis of dyadic coordination. Additionally, the game had a fully programmed feedback mechanism, which allowed for audible reinforcement throughout the game (i.e., animations produced after players completed the puzzle), without requiring outside input.

KASPAR the robot is an even more pronounced example of the ways that technology can supplement the role of the practitioner. In this study KASPAR took a human practitioner's place to motivate and guide two players to interact with one another, reducing the need for professionals and allowing for peer-directed play. Furthermore, though not directly compared to a human professional, KASPAR is unique in that he is a fully animated robot. The novelty of playing a game with KASPAR compared to a therapist likely enhanced the intervention's effects, particularly as research suggests children with ASC are more receptive to non-human social stimuli (Atherton and Cross, 2018). Indeed, the study showed that the players were more socially responsive in KASPAR's presence than when playing alone with their partner. This is a pertinent example of how novel technology stimulates players' imaginations and creates memorable social experiences that may offer advantages over more commonplace gaming platforms.

### Benefits and Areas of Improvement: Digital Games

Engineers who design complex games for use in ASC interventions, often in collaboration with autistic individuals and special educators, benefit from their ability to provide in-depth descriptions of the game development process. Such detail is undoubtedly of use to future developers hoping to build upon these advancements. Indeed, many of these games appear to be primarily developed to showcase what can be technologically possible in an ASC intervention. These technological innovations almost certainly offer improvements regarding an intervention's novelty and the player's immersive experience within the game. As autistic people report high levels of enjoyment when engaging with digital media (Gillespie-Lynch et al., 2014), it is not surprising that many game-based interventions rely on computerized technology. Digital games offer several advantages over analog games, including in-built performance tracking, more effortless customization, and improved visual engagement that may be particularly important for people with ASC.

However, what is gained with regard to novelty and innovation is often lost in the validity and viability of many of these digitalised games. Very few of the more technologically advanced games developed for people with ASC have formally tested their game effects. Instead, many of these studies have relied on anecdotal reports from parents, educators, or researchers or simply report what they expect repeated game-play would produce. While these programs are clearly at the forefront of technical innovation, testing the behavioral effects of digital gaming needs to be prioritized. This is particularly paramount with the aim to encourage more wide-spread adoption. As many of these games rely on expensive equipment (i.e., VR, digital touch tables, motion tracking devices, robots), investment in testing its suitability and efficacy is needed to justify everyday users investment.

To address these issues, engineers may want to include psychologists and educators in developing their programs to advise how targeted skills can be measured within the game and how these games can be situated into relevant curriculum. For instance, many of these games could involve built-in progress tracking, which shows how players changed their game-play strategies and behaviors over time. As these digital games can automatically store player data, developers should look at ways to analyse program logs to show functional improvements over time (i.e., increased frequency and complexity of interactions between co-players).

## Analog Games

### Example Analog Games

While technology is becoming more widely accessible, many innovative gaming technologies are still quite expensive and require more widespread access. However, there is no doubt that these innovative technologies will be more prominently featured in autism interventions in the coming years. A few are particularly promising and draw on the unique strengths of digital intervention and its benefits over analog games, to which we now turn.

Dell'Angela et al. (2020) offers a way to circumvent some of the challenges analog game studies present. In their study, the researchers devised a testing paradigm that tested how emotional competency measures correlated with in-game behaviors and perceived difficulty, and they compared game play across off the shelf and modified games. The video-coding and observational measures used in Baker (2000), Jung and Sainato (2015), and Daubert et al. (2015) revealed the nuanced changes in behavior that took place over many sessions. However, researchers with more limited resources and larger samples may want to utilize instead control groups or measures of within-subjects effects to assess behavioral change. As demonstrated by Dell'Angela et al. (2020), assessing how existing emotional and social competency measures relate to aspects of games that tap into these skills (i.e., perceived level of difficulty, points scored, number of games won) would allow for more sophisticated understanding of a game's cognitive effects. Taking such measures pre- and post-intervention could then be used to evaluate individual player's progress across an intervention (as was done in Teach Town).

Though it requires further formal testing, the studies focused on role-playing games in adolescent samples are quite promising with regards to their broader impact on social development. In Fein (2015) and Katō (2019) adolescents with ASC interacted with each other over an extended period of time, developing characters, and engaging in extended verbal interactions. Importantly, adolescents who participated had existing interests in these games, meaning that they had the opportunity to spend time with others who shared their enthusiasm and interests. Role-playing tabletop games may be particularly attractive to professionals who work with autistic adolescents as they encourage peers to form groups with other like-minded individuals, are readily available, and encourage independent social interaction between players. These games do not require professionals personalization as the games themselves require participants to create characters and self-directed narratives. Additionally, Fein (2015) suggested that autistic adolescents may feel more comfortable expressing themselves and experimenting socially by taking on a certain character in the game, and noted the positive culture that developed around role-playing games and autism acceptance. More formal research on the types of social interactions during role-playing games is warranted.

To move from research to practice, these two groups of researchers, i.e., those who specialize in game design and those who specialize in behavioral testing, should aim to blend their skill sets on joint ventures. In order for digital games to become widespread in professional settings, digital gaming prototypes should be more rigorously tested, particularly within the settings in which they are likely to be deployed (i.e., special schools and clinical practices). Care should also be taken that rich social interactions are not lost through increased attention to screens/graphics. Conversely, analog game interventions should be evaluated using less onerous testing procedures and should incorporate more sophisticated game designs that limit the need for outside support, and instead lead to peer-directed play. Understanding the unique benefits gained from digital vs. analog games should be comparatively assessed.

### Benefits and Areas of Improvement: Analog Games

While arguably not as vivid and immersive, what analog games may offer over and above digital games are that they are played in person and require face to face contact, which may improve the social connection between players. Though some digital games preserved the opportunity for face-to-face contact within their game design (Collaborative Puzzle, KASPAR), many digital games could conceivably lead players to focus more on the game rather than their play partner. As analog board games require players to physically face one another around a board and directly engage with one another verbally, they may offer the opportunity for richer social interactions than many digital games. As people with ASC are well-documented in their difficulties with in-person emotional recognition and communication, playing in-person games may allow them to practice these skills while simultaneously improving explicit social knowledge.

Understanding the rich social interactions that take place during face-to-face gaming interventions was the primary focus of many of the studies using analog games. In contrast to the programs that focus primarily on technologically advanced game development, many analog games discussed instead placed their efforts into testing the effects of pre-existing analog games (i.e., Wii, Candy Land, Bingo). Studies such as Baker (2000), Jung and Sainato (2015), and Daubert et al. (2015) all used off the shelf board games in their interventions, relying on small samples and single-case designs. Unlike the studies detailing the creation of complex technical programs, these interventions were relatively simple in their development. Instead, they prioritized tailoring these games to suit a particular autistic child's needs. They also used complex testing procedures to understand subtle behavioral changes over time (i.e., assessing baselines, video coding play behaviors across numerous sessions, creating reinforcement schedules).

As ASC is a highly heterogeneous condition, meaning that there is great variation in symptoms and abilities within the population, using more tailored protocols and assessments is a sensible approach. Additionally, studies such as Baker (2000), Jung and Sainato (2015), and Daubert et al. (2015) were particularly interested in the subtle social behaviors players exhibited in relation to other players (i.e., eye contact, reciprocity, and shared attention). Both stimulating and measuring these types of behavioral improvements, particularly with young children with limited verbal ability, can be difficult to induce and record without spending time with the child and relying on observational measures gathered at multiple time points. However, the time required to individualize existing games for each participant and manually code behaviors places high demands on professionals. It also limits the ability to replicate these interventions in larger groups and maintain the program for longer time-periods. Additionally, as these pre-existing games are not as complex and novel, they may require more adult input to keep players engaged in the intervention (i.e., Power Cards, video modeling, prompting, rewarding).

### Moving Forward

There are several areas of research that those interested in the effects of gaming on ASC may want to investigate in the future. First, it would be important to understand the effect that gamification in and of itself offers when employed in an autism intervention. Experimental studies that compare rates of behavioral change between groups who engage in game based vs. non-game based interventions are much needed. As games are meant to be intrinsically rewarding, measuring not only the effects of the game-based intervention has on particular skills, but also how it enhances the user experience, and how it socially engages a group of players, would be important areas of investigation.

Following on from this, it would be of interest to understand more about autistic user experience in relation to certain types of games. As highlighted by researchers who incorporated restricted interests into traditional board games, interacting with restricted interests can be particularly engaging and motivating for people with ASC. It would be of interest to test whether games that incorporate restricted interests boost participation and engagement. If so, it may be that some of the more common restricted interests could be incorporated into many different types of games, thus boosting player participation.

For older cohorts (adolescents and adults) researchers may want to investigate the social/hobbyist aspects of gaming and how this relates to ASC enthusiasts. Research suggests that autistic people are more likely to play video games, though less is understood about the relationship between autism and board gaming. Research into board and video game-play with adolescents and adults with ASC suggested that they experience increased quality of life and may have improved communication. This suggests that ASC friendly gaming communities may be a beneficial social outlet for those on the spectrum (Lancy and Grove, 2011).

While many studies offer promising results, it is clear from this review that more stringent testing procedures must be adopted to determine how effective many of these interventions are in naturalistic settings and how skills generalize over time.

As was also noted by Grossard et al. (2017), and what has also become apparent through this review, is that there appears to be a disconnect between two main research groups. Specifically, the aims of the engineers who design many of the cutting edge digital games, and the researchers who test pre-existing, more rudimentary games, are not always aligned.

These studies would particularly benefit from research into how educators, professionals, and families can readily implement them across settings (i.e., school, home, clinical practice). To aid professionals and families in deciding whether certain game-based interventions would meet their needs, a “ludography,” or game-based bibliography can be found in Table 3 where we focus specifically on the games used in these studies, detailing the player-experience, targeted outcome, and the necessary requirements for adopting such a program (i.e., materials, equipment). The primary purpose of this “ludography” or game-bibliography is to provide non-academic professionals who work with ASC individuals or parents of children with ASC a clear understanding of the choice of games available and the feasibility of implementing such a program. This table focuses specifically on the games used in ASC research and outlines necessary materials, equipment, and training to encourage more widespread adoption and validation of these approaches.

**Table 3:** “Ludography” detailing specific games tested on children with ASC and other conditions.

**Game (Reference)**	**Summary of game**	**Application**
Bingo (Baker, 2000)	Bingo involves children having a board that is delineated by rows and columns. A player wins Bingo by having a complete row/column that contains items announced by the Bingo caller. In this version, the Bingo game is modified to reflect an autistic child's restricted interests. For instance, a child who is interested in model cars, the bingo item is “called” by having the children launch cars off a track and note what car picture the model car lands upon following the jump. This picture must then match the picture on the Bingo card in order to be covered up.	Autistic children may be more receptive to learning games and playing with peers if games are centered around their preferred area of interest. To do this effectively, it is helpful for practitioners to learn about the child's interests from parents and create modifications to existing games for ease of access.
Recognition Game (Dell'Angela et al., 2020)	The Recognition Game is a variant of the game Mimtoo, a pantomime game in which children select at random a slip of paper with a sentence that they must act out for their team to guess. In this emotional competence version, the child who is set to pantomime will choose both a sentence and an emotion word which may or may not be congruent with the sentence (e.g., “My mother forgot my birthday” and one of the six emotions: happy, sad, frustrated, etc). Taking turns, each team will send a player to pantomime the sentence and act out the emotion. The goal is to guess as many correct emotions as possible in a given time.	This game could be a particularly engaging way to improve emotion recognition. The challenge is that the emotion may not match the sentence. However, it presents an interesting way about learning emotional context, as many sentences could be spoken in a different way to convey a different meaning. As children with ASC have difficulty interpreting emotions and understanding context in communication, it could be a particularly beneficial game to learn these two skills. Additionally, this game is played in a group setting that could improve peer relationships. Very little materials are needed to implement this game, making it very easy to adapt to various settings.
Differentiation Game (Dell'Angela et al., 2020)	The Differentiation Game is a variant of the game Codenames. Children in teams try to help their group guess a specific combination of word cards laid out on a grid based on a card identifying which words are safe and which are off limits. Players should only give clues that help identify certain words, and avoid clues leading to incorrect guesses. In this version, rather than choose any word as a clue, the player must use an emotional word.	In this version the skill being used is emotion recognition and the ability to perspective take. The team that is guessing the words must think about what the player likely feels about certain words on the board (i.e., “Do they get scared on roller coasters? Or maybe they find certain types of movies scary?”). This type of emotional reasoning is building on perspective-taking, while also encouraging children to think more broadly about emotions. This game allows for the adaptation of an existing game that is readily available, and refines it to target a specific emotional competency skill. It could also be used to help build relationships between team members, as they learn about one another by understanding what emotions a team member associates with certain words. This would be an excellent game to test on autistic children who are looking to build skills in this area.
Reappraisal Game (Dell'Angela et al., 2020)	The Reappraisal Game is a cooperative storytelling game in which children must incorporate randomly drawn story cards into a single narrative in sequential order. This game is based on the cooperative storytelling game Once Upon a Time. In this version of the game, there is an added layer to build emotional competencies. In this version, toward the end of the story one of the players draws a “complicator” card that introduces a negative element into the story. The player must then describe the emotions that the characters would experience following this complication. Another player designated to be the “optimist” must then roll a dice that matches a reappraisal strategy. They must then use that reappraisal strategy to see the complication's “bright side.” The other players then guess which reappraisal strategy had been used.	This game builds on several important cognitive skills. In line with the original version, the Reappraisal Game allows children to practice building a cohesive narrative and doing so with an audience in mind, a form of theory of mind. The Reappraisal game builds on this by requiring players to also focus on the story's emotional arc, first describing a complication and then a resolution. The children will be focusing on the resolution's emotional effects by identifying how the resolution was presented using cognitive reappraisal. This game may be beneficial for autistic children who experience difficulties in emotion recognition, narrative comprehension, and emotional regulation. Learning techniques for seeing the “bright side” of a negative event may be particularly helpful regarding this last skill. Like the last two games discussed in this study, this game would be easy to implement and suitable for many settings.
Linear Board Game (Satsangi and Bofferding, 2017)	In this game designed to build numerical competencies, the board, the children, played upon forms a number path, with 10 tiles numbered 1–10 displayed horizontally. The tiles alternated in color (red, blue, and green). To play the game, the children rolled a six-sided die, with half of the die faces numbered “1” and the other half “2.” The children then moved across the number line the number of spaces they had rolled, and verbally stated the numbers that they moved across on the board.	Children who played the game significantly improved in their numerical knowledge compared to those who had done a control condition using colors instead of numbers to advance across the board. This game is undoubtedly useful for children with autism who struggle with numerical understanding. However, it is rather rudimentary, and there doesn't appear to be a scoring system in place, which adds an element of competition or a reward for completion. If adopted by a professional it would be beneficial to add more complexities to the game, especially for older children. This is, however, an easy game to create and run for groups.
Power Card Games (Daubert et al., 2015)	Power Cards are behavioral reinforcement tools in which a child has a character related to their restricted interest model appropriate behaviors depicted on cards. These cards are then presented to the child during activities to remind the child of appropriate behaviors and to motivate them to emulate their favorite character. Power Cards were used to guide children through gameplay, specifically the games Topple, Operation, and Honey Bee Tree. All three of these games require players to use a similar motion, grasping, to reach a particular gaming goal. For instance, in the game Topple, players have to build the tallest structure they can without having the structure topple after placing a piece on the top. In these games, Power Cards were used to guide appropriate gameplay behavior, specifically initiating a turn, relinquishing a turn, and speaking encouragingly to other players.	Power Card techniques have been tested in other domains, and have improved behaviors in autistic children in a number of activities. This study suggests that they are particularly useful when introducing autistic children to gaming, and helping them play effectively with others. Power Cards are easy to adopt, as they require few materials, and are easy to adapt, as they follow a broad template designed to be customized to a child's favorite character. As autistic children may struggle with emotional regulation and reciprocity, they will likely need additional help when learning about good sportsmanship in gameplay. Using Power Cards within a gaming intervention may be particularly useful for those children who show particular difficulties with regard to these gaming skills.
Live Action Role Playing (LARPing) (Fein, 2015)	LARPing involves a group of participants create and enact a different story. In the LARPing activities discussed in the article below, the quest narratives were created by the Journeyfolk, who run a summer camp tailored for autistic adolescents in the United States. In LARPing the game is begun using a broad theme drawn from several different narratives. Each player then adds their own element to the story and their subsequent decisions within the game change the plot. Players develop characters that they embody within the game. These characters have backstories, personality traits, and customized costumes to fully realize their characters within the game.	In this qualitative study, the researcher found that autistic LARPers were empowered within the gaming event. They created meaning out of the quests that often appeared to express something about how they felt about themselves as an autistic person, including the challenges and the pride they felt in their autistic identity. Within the LARPing environment, campers had more structured social encounters that were made more clear by the constraints of the game. They were also able to engage with each other in creative ways by changing the narrative and personalizing their characters. Finally, campers formed a community with one another where they shared common interests and engaged with their autistic identity. Creating opportunities for LARPing among autistic adolescents may be a valuable endeavor. Such an intervention would be time consuming with regards to gaging interest and organizing the materials (i.e., scripts, costumes). However, if it stimulated excitement among participants it could lead to an autistic adolescent community where shared interests and acceptance of differences led to friendships and increased self-esteem.
Table Top Role Playing Games (Katō, 2019)	Interactive games in which a small group of players interact through a fictional story setting. Using pencils, paper, and dice, TRPG players explore their character's personality, background, and goals to construct the story with other players in the form of in-game role playing. Their choices within the game will affect the outcome of the group. TRPGs have been found to enhance creativity and divergent thinking and have been used therapeutically to build social skills and self-esteem in teens and young adults.	For autistic teens with typically developed verbal ability, training-based communication support, which teaches routine methods for conversation, may not provide naturalistic opportunities for communication. They also rely heavily on professional help rather than learning through everyday interactions with peers. Creating opportunities for autistic teens to interact with peers in an engaging environment may teach communication skills absent from stricter communication training programs. The games themselves may be intrinsically motivating as they allow for creativity, character development, and immersion in the game.
Speech Therapy Games Hoque et al. (2009)	Players are completing traditional speech therapy activities in the context of a computer game. For instance, in the game, a participant would be required to modulate the volume of their speech, or the rate of their speech, to control objects in the game. The authors provide limited information about this game, and therefore the description above is brief.	There is not enough information about the game to say whether it offers definitive improvements over traditional speech therapy with a speech and language specialist. However, the preliminary results suggested that this was more engaging to students than the traditional delivery. The program appears to be easy to implement as the only necessary equipment would be a computer and a microphone headset. However, it is unclear whether this program is readily available for use or purchase. Professionals who wish to explore this option can contact the authors for more information.
Candy Land (Jung and Sainato, 2015)	Candy Land is a board game in which players draw cards with either colors or characters. Players then move their game pieces along the Candy Land board using the cards as guidance. Various challenges within the game allow some players to advance a greater distance at specific points, or get stuck. Players win when they reach the end of the board. There are special editions of Candy Land. In this study, a young autistic girl who was interested in princesses played the Candy Land: Disney Princesses version of the game.	Candy Land was supplemented by a video modeling program in which instructors recorded videos of themselves playing the game dressed as a princess. The child then dressed up as the princess to play the game. This was done to encourage the child to model their behavior after the video, and to maintain interest in the game theme. The behaviors targeted related to engagement, social reciprocity, and reducing inappropriate behavior. Results suggested that not only did behaviors improve in the game, but they generalized to a new game without the embedded character. This intervention would be somewhat lengthy to implement as it would involve video recording behaviors as certain characters. However, this may be necessary for children with ASC who would not otherwise be motivated to participate. That this technique led to some generalization to novel games is encouraging. Though the process of recording the videos for each child may be time consuming, it is something that would be readily accessible i.e., no specialist tools are required.
Make n Break (Jung and Sainato, 2015)	Make n Break is a game in which players must follow a blueprint to build a certain structure using 10 colorful wooden blocks. Players must complete as many of the 60 structures as they can in the allotted time indicated on the dice, which they roll prior to beginning construction. In this study cited below, Make N Break was combined with the video modeling approach described in the above game.	Similar to the previous discussion of the game Candy Land and how it is combined with video modeling, this seems to be a valuable tool to increase engagement and appropriate gaming behavior in autistic children. While it is not a ready-made intervention, meaning a professional will need to create customized videos demonstrating gaming behavior in line with the child's interests, it significantly increases motivation to play games. Professionals may want to explore this option to improve social functioning in autistic children.
Pico's Adventure (Malinverni et al., 2017)	Pico's Adventure is a Kinect based game in which children played a whole-body video game over four sessions. In the game, children go on a series of adventures to help Pico, an alien, complete different missions (i.e., fixing his spaceship). In the four sessions, the children work on basic social initiation, cooperation, joint attention, and turn-taking. In the first session, the child meets the character and familiarizes themselves with the environment. In the subsequent sessions the child plays in tandem with a parent/professional and another autistic child. While the intervention's effects were not formally tested with regards to improvements on the target behaviors, an exploratory study found that the children readily engaged in the task and were eager to explore the digital environment.	Pico's Adventure could be a fun addition to a social skills curriculum for ASC children, either at school or at home. Kinect equipment is needed, but this is readily available for purchase. One of Pico's Adventure's strengths is that it was designed with the help of ASC professionals and children, making it particularly appealing to its target demographic. As it encourages whole-body movement and encourages social interaction between two players, it could be a stimulating way to improve reciprocal interactions between peers, though this needs more formal testing. Pico's Adventure also includes a narrative, in that children are learning about Pico throughout the game and helping him return to his planet after accidentally landing on earth. It would be interesting to explore whether narrative comprehension is also positively affected following the intervention. Professionals or parents looking to engage children with ASC in a social intervention that may also improve narrative comprehension may want to include this game in a curriculum.
• Consoles Wii (i.e., baseball/wiffle/sports) • Kinect (Ferguson et al., 2013) • Edwards et al. (2017)	• Nintendo Wii is a video game console that allows for motion-controlled gaming. Wii has motion sensing technologies and a Wii remote, which can be used as a pointing device or as a means to detect whole body or arm motion. The Wii console is readily available and has games suitable for all ages. • Xbox Kinect is also a motion-sensing video game console. Unlike the Wii which uses a handheld remote, the Kinect uses cameras and microphones to allow the device to recognize speech and detect the bodies of up to four players. The Kinect camera sits at the top of the user's display and operates like a webcam. • Both consoles allow users to play games that allow the user to interact within the gaming environment using whole-body motion to mimic real-life gameplay (i.e., hitting a virtual tennis ball by swinging the arm in real-time).	• In the two papers listed, Wii and Kinect were used to encourage autistic children to play virtual sports games and, in the process, improve their sportsmanship or their object control abilities. Both interventions showed an improvement, either in certain sportsmanship behaviors or in perceived sports abilities. This suggests that both gaming platforms can teach new behaviors and improve self-perceptions of physical competence. Both platforms allow for autistic children to interact with other players through an engaging virtual environment. As both platforms allow for multiple users, they would be a useful addition to curriculums aiming to improve social skills and/or physical activity as they encourage whole-body movement and social interaction, either competitively or cooperatively. • Both consoles are similarly priced. However, Wii games require an additional remote for each player, and motion tracking is only done through the handheld remote. The Kinect can track whole-body movement (i.e., arms legs head) and has voice recognition. As it does not require a remote, up to four players can be involved without any additional equipment. The Kinect, however, requires the Xbox console in addition to the Kinect, so it is more expensive. However, if it is possible, the Kinect may be more suitable for autistic children who have more significant needs as it does not require holding a remote and is more sensitive with regards to movement tracking. For interventions in which practitioners want to understand motor differences in autistic children, the Kinect will offer a more fine-grained analysis. The Kinect can also project the user's image into the game, particularly engaging players.
Collaborative Puzzle Game (Battocchi et al., 2009)	The Collaborative Puzzle Game (CPG) is a two-player puzzle game played on a touch screen tabletop. In the game, players must complete jigsaw puzzles in tandem. Specifically, to have a piece move, both players must be dragging it together simultaneously with their fingertips. During the puzzle completion phase and after completing a puzzle, the users are given visual and auditory feedback that alerts them if a mistake is made and gives them praise for having finished the puzzle. The equipment needed for the game is an interactive touch screen tabletop.	• Research on the CPG showed that while the interactions with players who had to move the puzzle pieces in tandem with one another were more complex and completion time was longer, collaboration was higher than if the puzzle was completed independently. Players were also more coordinated with one another in the collaborative condition. Thus, it appears that the CPG is a useful tool for encouraging social interactions between children with ASC and may even lead to higher degrees of problem-solving as the puzzle was more difficult and communication more nuanced. • The CPG and its delivery using a touch screen table offer several benefits due to its unique format. The touch screen allows for a more naturalistic and intuitive way to complete a puzzle, in line with real-life jigsaw puzzle motions. The fact that it is delivered on a tabletop rather than a tablet, for instance, means that players have to coordinate with their bodies (i.e., leaning over the table, moving around the table), which could help autistic children who have motor difficulties. As this is a digitized puzzle, it can be programmed to deliver prompting and reinforcement, and can change the level of difficulty and the requirements for success (not allowing puzzle piece movement unless two children are touching the piece). This offers advantages over non-computerized tasks of a similar nature. However, purchasing a digital tabletop will not be feasible for everyone. It may, however, be a useful addition to a school or larger facility with more resources. However, unlike more popular consoles like the Kinect or Wii it is not clear how many programs are available on the digital touch table. Activities for the device may be more limited.
Lets Face It (Tanaka et al., 2010)	Lets Face It (LFI) is a computer game intervention in which children practice various face recognition skills. Specifically, LFI comprises seven games that target facial identification aspects, including holistic face processing, memory for faces, facial expression, and face dimensions. The intervention takes 20 h to complete, with a recommended time of 100 min spent playing LFI per week. LFI has built-in rewards and incentives, including a high score table and animated graphics. Children are also able to select the mode and level of gameplay. The LFI also has a face recognition battery test delivered over the computer, allowing for an understanding of baseline and post-treatment face recognition abilities. This program only requires a computer, as the program is available for free.	Results from the study listed below showed that LFI improved some aspects of face recognition in children with ASC, including recognition of the eyes and the mouth. However, most subtests did not see substantial improvement following the intervention. Additionally, this program did not assess whether real-life improvements in face recognition occurred following the intervention. As this program is available free of charge and requires little equipment, it may be a viable option for parents or professionals interested in targeting facial recognition skills in autistic children. However, this intervention only allows for independent gameplay. As many of the other games target specific skills while also allowing for peer-directed play, LFI may not be as effective in simultaneously teaching children about reciprocal social interactions or help with relationships development with peers. It may be that LFI is most useful in conjunction with a real-life peer activity (i.e., a role-playing game or a board game) where children can practice the facial recognition skills they learned in the game in real-life situations.
TeachTown (Whalen et al., 2010)	TeachTown is a computer-delivered intervention targeting academic and social skills. Specifically, the program targets receptive language, social understanding, life skills and cognition/academics. TeachTown is delivered through 20-min sessions over the computer and with an educator every day for 3 months. The program uses the basic principles of ABA. Specifically, children are encouraged to supply correct responses by receiving the opportunity to play games after they correctly complete a task. Children progress through at their own pace. After they master a lesson they move onto new material; if they do not progress on a pre-test then they are given training on the material until they master the content. In the in-person lessons provided by their instructor to the whole class, the teacher implements activities that target skills not included in the TeachTown curriculum (i.e., imaginative play, daily living skills).	Children who completed Teach Town showed significant improvement on the program's lessons. Though not significant, they had higher scores than waitlisted children on standardized assessments of the four TeachTown skill categories. This curriculum may be of interest to special educators interested in incorporating a computer-based intervention. TeachTown uses ABA principles, it may be particularly effective for classrooms that already use these techniques. More research is needed on the real life effects of TeachTown and the long term effects of the intervention. As this is delivered to young children, it may be that improvements following Teach Town are more obvious in following years, though this has yet to be tested. As the intervention must be completed daily, it may be most feasible for schools with multiple available computers so that several children can complete the tasks simultaneously. This may not be suitable for all children as it requires the ability to use a mouse and attend to the computer for 20 min.
KASPAR (Wainer et al., 2014)	In this intervention, two children interact with a humanoid robot, KASPAR, while playing an imitation game. KASPAR appears similar to a baby doll, with realistic skin and hair, and has an animated face and free moving arms. KASPAR is able to speak and change his facial expressions. In the game, two children use Nintendo Wii controllers to complete a mimicry challenge similar to the game Simon Says. Specifically, one child would pose in a specific way by following a screen in front of them showing a specific pose depicted by a stick figure. The child would then strike that pose and communicate to their partner how to copy the pose. KASPAR's role in the game was to provide verbal encouragement, reminders and to be a third player in the imitation game. The equipment needed for this intervention is an animated robot doll and a digitized version of Simon Says.	Children who played together alongside KASPAR were more animated and showed more positive affect toward one another. At the same time, when playing with KASPAR, participants were less successful at the imitation task. Nonetheless, it appears that KASPAR improves social interactions between children with ASC and is an engaging addition to game play. KASPAR is not readily available for purchase, so this intervention's usability is limited. Additionally, though Wii remotes were used in the imitation game tested in this project, the imitation game is also not readily available. Specialists wishing to include a robot intervention may want to explore other more readily available options. For instance, many games have on-screen avatars which provide reinforcement similar to KASPAR. Researchers may want to test whether the inclusion of an avatar and the creation of an imitation game on a console like Wii or Kinect can produce similar effects.
ADDventourous Rhythmic Planet (Giannaraki et al., 2019)	In this virtual reality (VR) game, players use a drum to create a rhythm. This drumming is then turned into a gaming action that is visually represented in the VR space. In the game, the hero is an alien who only progresses if a player reproduces a rhythm correctly, allowing the alien to continue its journey into the game's next stage. The levels become increasingly difficult. The game comes with two modes, single and multi-user. The game plot encourages children to play with one another and move from single to multiplayer mode. When playing in multiplayer mode, the rhythm is created collaboratively. This game requires VR headsets, a drum that can transmit to the VR system, and computing systems that are able to run the VR platform Unity.	This game offers an engaging, multi-sensory digital environment in which children are encouraged to create music with one another and, in doing so, receive in-game rewards and complete a narrative quest. This program uses state of the art VR technology to create a 3-d visual game, thus providing a highly immersive gaming experience. Though originally designed with children with ADHD in mind, this would likely be beneficial to autistic children as well, as it encourages joint action between peers and helps build rhythmic competencies, which are often found to be disrupted. One of the limitations to this program is the feasibility and lack of formal testing. VR systems are expensive and highly technical. They require training with regards to set-up and testing, and may not be suitable for professionals, educators or parents. Additionally, while the VR systems are available for purchase, the intervention's drum to guide gameplay is not. Those wishing to use this intervention would have to create a similar instrument on their own. Researchers may want to investigate ways to recreate this game using less expensive, more readily available equipment and perhaps use an alternative to the physical drum (i.e., using a remote that simulates a drumstick to provide a similar movement). Finally, the effects of the intervention were not tested. More research is needed to determine how this improves skills in children with disabilities.
Magic Mat (Politopoulos et al., 2021)	In this video game, users stand on a mat that can track movement and thus guide on screen actions. Magic Mat is analogous to a large keyboard on the floor; it has arrows that the user steps on that guide the on-screen movements. For instance, in the game Tetris a user must guide falling blocks to angle them into gaps with a similar shape on the bottom of the screen. Using Magic Mat, the user does this by stepping on the appropriate arrow rather than sitting on a computer and doing this on the keyboard. This format encourages the user to use whole body movement to play traditional computer games. This requires a Magic Mat and compatible games in order to be used.	Though this was not designed specifically for ASC, it is relevant in that autistic children show an interest in computer games and struggle with coordination. The Magic Mat approach to video games may encourage more whole body movement, improved spatial awareness and even collaboration between multiple players. Magic Mat is a gaming prototype and is thus not readily available. However, it is a rather simplistic device that could likely be created by individuals with computer programming ability. It would be interesting for future research to test the effects of Magic Mat on autistic children with regards to physical coordination and peer collaboration. At present, however, it needs more testing and development in order to be used by professionals and families.

In conclusion, there are many games that have been developed as autism interventions, many of which are discussed in this scoping review. These gamified interventions target three key areas: socio-communication skills, academic skills, and physical skills. While many games have been developed, few have been tested with large samples and many have not shown how skills improved or whether they generalize to other settings. Digital games show promise in that they are designed to maximize engagement and reinforcement, but they need to be formally tested to see whether improvements in the game extend to improvements in real life. Off the shelf board games that have been adapted for autistic players should be tested on larger samples and would benefit from easier data collection processes and more sophisticated ways to customize them to individual players. The most effective and accessible interventions reviewed were those that encouraged interactions between players, and used simpler game designs. Researchers who specialize in behavioral testing, and those who specialize in gaming innovations will want to collaborate in future on gaming interventions that are both efficacious and innovative. More broadly, researchers should investigate the effects of gamification on autism interventions more generally, particularly as motivation as it relates to ASC is not yet fully understood.

## Author Contributions

All authors listed have made a substantial, direct and intellectual contribution to the work, and approved it for publication.

## Conflict of Interest

The authors declare that the research was conducted in the absence of any commercial or financial relationships that could be construed as a potential conflict of interest.

## Publisher's Note

All claims expressed in this article are solely those of the authors and do not necessarily represent those of their affiliated organizations, or those of the publisher, the editors and the reviewers. Any product that may be evaluated in this article, or claim that may be made by its manufacturer, is not guaranteed or endorsed by the publisher.

## References

[B1] Abirached B. Yan Z. Aggarwal J. K. Tamersoy B. Fernandes T. Miranda J. C.. (2011). Improving communication skills of children with ASDs through interaction with virtual characters, Paper presented at the 2011 IEEE 1st International Conference on Serious Games and Applications for Health (SeGAH) (Washington, DC).

[B2] American Psychiatric Association (2013). Diagnostic and Statistical Manual of Mental Disorders (DSM-5®). Washington, DC: American Psychiatric Pub.

[B3] Asperger H. (1944). “Die Autistischen Psychopathen” im Kindesalter. Arch. Psychiatr. Nervenkrankh. 117, 76–136.

[B4] Atherton G. Cross L. (2018). Seeing more than human: autism and anthropomorphic theory of mind. Front. Psychol. 9:528. 10.3389/fpsyg.2018.00528 29755383 PMC5932358

[B5] Baker M. J. (2000). Incorporating the thematic ritualistic behaviours of children with autism into games: increasing social play interactions with siblings. J. Posit. Behav. Intervent. 2, 66–84. 10.1177/109830070000200201

[B6] Battocchi A. Pianesi F. Tomasini D. Zancanaro M. Esposito G. Venuti P.. (2009). Collaborative Puzzle Game: a tabletop interactive game for fostering collaboration in children with Autism Spectrum Disorders (ASD), Paper Presented at the Proceedings of the ACM International Conference on Interactive Tabletops and Surfaces (Banff, AB). 10.1145/1731903.1731940

[B7] Behnamghader M. Khaleghi A. Izadpanah P. Rahmani F. (2019). Using gamification based on mobile platform in therapeutic interventions for children with dyslexia, in Internet of Things, Infrastructures and Mobile Applications. IMCL 2019. Advances in Intelligent Systems and Computing, eds Auer M. E. Tsiatsos T. (Cham: Springer), 814–824.

[B8] Bernardini S. Porayska-Pomsta K. Smith T. J. (2014). ECHOES: An intelligent serious game for fostering social communication in children with autism. Inf. Sci. 264, 41–60. 10.1016/j.ins.2013.10.027

[B9] Brown J. Murray D. (2001). Strategies for enhancing play skills for children with autism spectrum disorder. Educ. Train. Ment. Retard. Dev. Disabil. 36, 312–317.

[B10] Carvalho V. H. Brandão J. Cunha P. Vasconcelos J. Soares F. (2015). Tobias in the zoo – a serious game for children with autism spectrum disorders. Int. J. Adv. Corp. Learn. 8, 23–29. 10.3991/IJAC.V8I3.4897

[B11] Chamberlain B. Kasari C. Rotheram-Fuller E. (2007). Involvement or isolation? The social networks of children with autism in regular classrooms. J. Autism Dev. Disord. 37, 230–242. 10.1007/s10803-006-0164-4 16855874

[B12] Chevallier C. Kohls G. Troiani V. Brodkin E. S. Schultz R. T. (2012). The social motivation theory of autism. Trends Cogn. Sci. 16, 231–239. 10.1016/j.tics.2012.02.007 22425667 PMC3329932

[B13] Cross L. Turgeon M. Atherton G. (2019). How moving together binds us together: the social consequences of interpersonal entrainment and group processes. Open Psychol. 1, 273–302. 10.1515/psych-2018-0018

[B14] Daubert A. Hornstein S. Tincani M. (2015). Effects of a modified power card strategy on turn taking and social commenting of children with autism spectrum disorder playing board games. J. Dev. Phys. Disabil. 27, 93–110. 10.1007/s10882-014-9403-3

[B15] Dautenhahn K. Billard A. (2002). Games children with autism can play with robota, a humanoid robotic doll, in Universal Access and Assistive Technology London, eds Keates S. Langdon P. Clarkson P. J. Robinson P.

[B16] Davis M. Otero N. Dautenhahn K. Nehaniv C. L. Powell S. D. (2007). Creating a software to promote understanding about narrative in children with autism: reflecting on the design of feedback and opportunities to reason, Paper Presented at the 2007 IEEE 6th International Conference on Development and Learning (London).

[B17] Davis-Temple J. Jung S. Sainato D. M. (2014). Teaching young children with special needs and their peers to play board games: effects of a least to most prompting procedure to increase independent performance. Behav. Anal. Pract. 7, 21–30. 10.1007/s40617-014-0001-8 27019792 PMC4711729

[B18] Dell'Angela L. Zaharia A. Lobel A. Vico Begara O. Sander D. Samson A. C. (2020). Board games on emotional competences for school-age children. Games Health J. 9, 187–196. 10.1089/g4h.2019.0050 32053027

[B19] Eckerman C. O. Stein M. R. (1990). How imitation begets imitation and toddlers' generation of games. Dev. Psychol. 26, 370–378. 10.1037/0012-1649.26.3.370

[B20] Edwards J. Jeffrey S. May T. Rinehart N. J. Barnett L. M. (2017). Does playing a sports active video game improve object control skills of children with autism spectrum disorder? J. Sport Health Sci. 6, 17–24. 10.1016/j.jshs.2016.09.004 30356508 PMC6188903

[B21] Fein E. (2015). Making meaningful worlds: role-playing subcultures and the autism spectrum. Cult. Med. Psychiatry 39, 299–321. 10.1007/s11013-015-9443-x 25812848 PMC4457285

[B22] Ferguson B. R. Gillis J. M. Sevlever M. (2013). A brief group intervention using video games to teach sportsmanship skills to children with autism spectrum disorders. Child Fam. Behav. Ther. 35, 293–306. 10.1080/07317107.2013.846648

[B23] Filsecker M. Hickey D. T. (2014). A multilevel analysis of the effects of external rewards on elementary students' motivation, engagement and learning in an educational game. Comput. Educ. 75, 136–148. 10.1016/j.compedu.2014.02.008

[B24] Gallup J. Serianni B. Duff C. Gallup A. (2016). An exploration of friendships and socialization for adolescents with autism engaged in massively multiplayer online role-playing games (MMORPG), Education and Training in Autism and Developmental Disabilities (Arlington, TX), 223–237.

[B25] Gaudi G. Kapralos B. Uribe-Quevedo A. Hall G. Parvinchi D. (2019). Autism Serious Game Framework (ASGF) for Developing Games for Children with Autism, in Interactive Mobile Communication, Technologies and Learning, eds Auer M. E. Tsiatsos T. (Cham: Springer), 3–12.

[B26] Giannaraki M. Moumoutzis N. Kourkoutas E. Mania K. (2019). ADDventurous rhythmical planet: a 3D rhythm-based serious game for social skills development of children with ADHD, Paper Presented at the Interactive Mobile Communication, Technologies and Learning (Thessaloniki).

[B27] Gillespie-Lynch K. Kapp S. K. Shane-Simpson C. Smith D. S. Hutman T. (2014). Intersections between the autism spectrum and the internet: Perceived benefits and preferred functions of computer-mediated communication. Intellect. Dev. Disabil. 52, 456–469. 10.1352/1934-9556-52.6.456 25409132

[B28] Gowen E. (2012). Imitation in autism: why action kinematics matter. Front. Integr. Neurosci. 6:117. 10.3389/fnint.2012.00117 23248591 PMC3521151

[B29] Grossard C. Grynspan O. Serret S. Jouen A.-L. Bailly K. Cohen D. (2017). Serious games to teach social interactions and emotions to individuals with autism spectrum disorders (ASD). Comput. Educ. 113, 195–211. 10.1016/j.compedu.2017.05.002

[B30] Hiniker A. Daniels J. W. Williamson H. (2013). Go go games: therapeutic video games for children with autism spectrum disorders, in Proceedings of the 12th International Conference on Interaction Design and Children (New York, NY), 463–466. 10.1145/2485760.2485808

[B31] Hoque M. Lane J. Kaliouby R. Goodwin M. Picard R. (2009). Exploring speech therapy games with children on the autism spectrum, in 10th Annual Conference of the International Speech Communication Association, INTERSPEECH 2009 (Brighton).

[B32] Jung S. Sainato D. M. (2015). Teaching games to young children with autism spectrum disorder using special interests and video modelling. J. Intell. Dev. Disabil. 40, 198–212. 10.3109/13668250.2015.1027674

[B33] Kanner L. (1943). Autistic disturbances of affective contact. Nerv. Child 2, 217–250. 4880460

[B34] Katō K. (2019). Employing tabletop role-playing games (TRPGs) in social communication support measures for children and youth with autism spectrum disorder (ASD) in Japan: a hands-on report on the use of leisure activities. Jpn. J. Analog Role Playing Game Stud. 23–28.

[B35] Khaleghi A. Heydari F. Takhttavani M. Haedar H. Soltaninezhad A. (2019). Combined approach to diagnose ADHD: gamifying conners rating scale, in Interactive Mobile Communication, Technologies and Learning, eds Auer M. E. Tsiatsos T. (Cham: Springer), 825–835.

[B36] Kim B. Lee D. Min A. Paik S. Frey G. Bellini S.. (2020). PuzzleWalk: A theory-driven iterative design inquiry of a mobile game for promoting physical activity in adults with autism spectrum disorder. PLoS ONE 15:e0237966. 10.1371/journal.pone.0237966 32911501 PMC7482920

[B37] Klopotova E. E. Krupnova I. Y. (2020). Possibilities of using board games to develop communication skills in children with autism spectrum disorder. Bull. Psychol. Pract. Educ. 17, 41–50. 10.17759/bppe.2020170105

[B38] Lancy D. F. Grove M. A. (2011). Marbles and Machiavelli: the role of game play in children's social development. Amer. J. Play 3, 489–499.

[B39] Li B. Atyabi A. Kim M. Barney E. Ahn A. Y. Luo Y.. (2018). Social influences on executive functioning in autism: design of a mobile gaming platform, in Proceedings of the 2018 CHI Conference on Human Factors in Computing Systems (Montreal, QC), 1–13. 10.1145/3173574.3174017

[B40] Malinverni L. Mora-Guiard J. Padillo V. Valero L. Hervás A. Pares N. (2017). An inclusive design approach for developing video games for children with Autism Spectrum Disorder. Comput. Hum. Behav. 71, 535–549. 10.1016/j.chb.2016.01.018

[B41] Marsh K. L. Isenhower R. W. Richardson M. J. Helt M. Verbalis A. D. Schmidt R. C.. (2013). Autism and social disconnection in interpersonal rocking. Front. Integrat. Neurosci. 7:4. 10.3389/fnint.2013.00004 23423608 PMC3575023

[B42] Mazurek M. O. Engelhardt C. R. Clark K. E. (2015). Video games from the perspective of adults with autism spectrum disorder. Comput. Hum. Behav. 51, 122–130. 10.1016/j.chb.2015.04.06232419043

[B43] Mercado J. Espinosa-Curiel I. Escobedo L. Tentori M. (2019). Developing and evaluating a BCI video game for neurofeedback training: the case of autism. Multimed. Tools Appl. 78, 13675–13712. 10.1007/s11042-018-6916-2

[B44] Milley A. Machalicek W. (2012). Decreasing students' reliance on adults: a strategic guide for teachers of students with autism spectrum disorders. Intervent. School Clin. 48, 67–75. 10.1177/1053451212449739

[B45] Milton D. E. (2012). On the ontological status of autism: the ‘double empathy problem’. Disabil. Soc. 27, 883–887. 10.1080/09687599.2012.710008

[B46] Murdock L. C. Ganz J. Crittendon J. (2013). Use of an iPad play story to increase play dialogue of preschoolers with autism spectrum disorders. J. Autism Dev Disord. 43, 2174–2189. 10.1007/s10803-013-1770-6 23371509

[B47] Narimani A. Khaleghi A. Haedar H. Semnani F. (2019). The use of gamification in evaluating children's emotional intelligence, in Interactive Mobile Communication, Technologies and Learning, eds Auer M. E. Tsiatsos T. (Cham: Springer), 806–813.

[B48] Noda S. Shirotsuki K. Nakao M. (2019). The effectiveness of intervention with board games: a systematic review. BioPsychoSoc. Med. 13:22. 10.1186/s13030-019-0164-1 31641371 PMC6802304

[B49] Oppenheim-Leaf M. L. Leaf J. B. Dozier C. Sheldon J. B. Sherman J. A. (2012). Teaching typically developing children to promote social play with their siblings with autism. Res. Autism Spectr. Disord. 6, 777–791. 10.1016/j.rasd.2011.10.010

[B50] Piaget J. (1997). The Moral Judgement of the Child. New York, NY: Simon and Schuster.

[B51] Pliasa S. Fachantidis N. (2019). Can a robot be an efficient mediator in promoting dyadic activities among children with Autism Spectrum Disorders and children of Typical Development?, in Proceedings of the 9th Balkan Conference on Informatics (Sofia), 1–6. 10.1145/3351556.3351592

[B52] Politopoulos N. Stylianidis P. Apostolidis I. Chaldogeridis A. Mavropoulou A. Tsiatsos T. (2021) Creating magic-matt, an interface to transform video games to a sports experience, in Internet of Things, Infrastructures Mobile Applications. IMCL 2019. Advances in Intelligent Systems Computing, Vol. 1192, eds Auer M. E. Tsiatsos T. (Cham: Springer). 10.1007/978-3-030-49932-7_59

[B53] Politopoulos N. Stylianidis P. Apostolidis I. Chaldogeridis A. Mavropoulou A. Tsiatsos T. (2019). Creating magic-matt, an interface to transform video games to a sports experience, in Interactive Mobile Communication, Technologies and Learning (Springer, Cham), 629–637.

[B54] Rogerson M. J. Gibbs M. R. Smith W. (2018). Cooperating to compete: the mutuality of cooperation and competition in boardgame play, Paper Presented at the Proceedings of the 2018 CHI Conference on Human Factors in Computing Systems (Montreal, QC). 10.1145/3173574.3173767

[B55] Satsangi R. Bofferding L. (2017). Improving the numerical knowledge of children with autism spectrum disorder: the benefits of linear board games. J. Res. Spec. Educ. Needs 17, 218–226. 10.1111/1471-3802.12380

[B56] Silva-Calpa G. F. M. Raposo A. B. Suplino M. (2018, May). CoASD: a tabletop game to support the collaborative work of users with autism spectrum disorder, in 2018 IEEE 6th International Conference on Serious Games and Applications for Health (SeGAH) (Vienna: IEEE), 1–8. 10.1109/SeGAH.2018.8401358

[B57] Tanaka J. W. Wolf J. M. Klaiman C. Koenig K. Cockburn J. Herlihy L.. (2010). Using computerized games to teach face recognition skills to children with autism spectrum disorder: the Let's Face It! program. J. Child Psychol. Psychiatry 51, 944–952. 10.1111/j.1469-7610.2010.02258.x 20646129

[B58] Tanner J. E. Byrne R. W. (2010). Triadic and collaborative play by gorillas in social games with objects. Anim. Cogn. 13, 591–607. 10.1007/s10071-009-0308-y 20066451

[B59] Trevarthen C. Daniel S. (2005). Disorganized rhythm and synchrony: early signs of autism and Rett syndrome. Brain Dev. 27(Suppl. 1), S25–S34. 10.1016/j.braindev.2005.03.016 16182487

[B60] Wainer J. Robins B. Amirabdollahian F. Dautenhahn K. (2014). Using the Humanoid robot KASPAR to autonomously play triadic games and facilitate collaborative play among children with autism. IEEE Trans. Auton. Ment. Dev. 6, 183–199. 10.1109/TAMD.2014.2303116

[B61] Whalen C. Moss D. Ilan A. B. Vaupel M. Fielding P. Macdonald K.. (2010). Efficacy of TeachTown: basics computer-assisted intervention for the intensive comprehensive autism program in Los Angeles unified school district. Autism 14, 179–197. 10.1177/1362361310363282 20484002

[B62] Wong C. Kasari C. (2012). Play and joint attention of children with autism in the preschool special education classroom. J. Autism Dev. Disord. 42, 2152–2161. 10.1007/s10803-012-1467-2 22350340 PMC4205103

[B63] Zakari H. M. Ma M. Simmons D. (2014). A review of serious games for children with autism spectrum disorders (ASD), in International Conference on Serious Games Development and Applications, (Cham: Springer), 93–106.

